# The Sound Sensitivity Symptoms Questionnaire Version 2.0 (SSSQ2) as a Screening Tool for Assessment of Hyperacusis, Misophonia and Noise Sensitivity: Factor Analysis, Validity, Reliability, and Minimum Detectable Change

**DOI:** 10.3390/brainsci15010016

**Published:** 2024-12-27

**Authors:** Hashir Aazh, Fatma Betul Kula

**Affiliations:** 1 Research and Development Department, Hashir International Specialist Clinics & Research Institute for Misophonia, Tinnitus and Hyperacusis Ltd., 167-169 Great Portland Street, London W1W 5PF, UK; info@hashirtinnitusclinic.com; 2 Department of Psychology, University of Surrey, Guildford GU2 7XH, UK

**Keywords:** psychometry, hyperacusis, misophonia, noise sensitivity

## Abstract

**Background/Objectives:** The Sound Sensitivity Symptoms Questionnaire version 2 (SSSQ2) is a brief clinical tool with six items designed to be used (1) as a measure for severity of sound sensitivity symptoms in general (based on its total score) and (2) as a checklist to screen different forms of sound sensitivity. The objective of this study was to assess the psychometric properties of the SSSQ2. **Method:** This was a cross-sectional study. A total of 451 people completed the online survey. A total of 154 people completed the survey twice with a two-week interval to establish test–retest reliability. The average age of the participants was 36.5 years (range 18 to 86 years). **Results:** Confirmatory factor analysis showed that the SSSQ2 is a one-factor questionnaire. Cronbach’s *α* was 0.80. The test–retest reliability was good for the total SSSQ2 score and was moderate for the sum of items 1 and 3 (indicating loudness hyperacusis), item 2 (for pain hyperacusis), item 4 (for misophonia), item 5 (for fear hyperacusis), and item 6 (for noise sensitivity). The minimum amount of change that constitutes a true change in the total SSSQ2 score is ≥5 points. **Conclusions:** The SSSQ2 can be used in clinical practice or research setting to measure the severity of general sound sensitivity as a one-factor questionnaire with acceptable internal consistency and good reliability. In addition, the individual items in the SSSQ2 can be used as a checklist to screen for various forms of sound sensitivity.

## 1. Introduction

Sound sensitivity, in this paper, is used as an umbrella term encompassing various disorders of sound intolerance ranging from hyperacusis to misophonia and noise sensitivity. Hyperacusis is the perception of certain everyday sounds, such as domestic noise or noise in public places, as too loud or painful in such a way that it causes significant distress and impairment in social, occupational, recreational, and other day-to-day activities [1,2]. There are several forms of hyperacusis (i.e., loudness, pain, and fear hyperacusis) [3]. Loudness hyperacusis refers to perception of certain everyday sounds (e.g., people’s voices, traffic noise, music, wind noise, hand dryers, hair dryers, door slamming, dogs barking, and the noise made by household appliances) as uncomfortably loud [4,5]. Individuals with loudness hyperacusis may also experience certain bodily sensations (e.g., aural fullness, pressure in ears, spasm/flutter in the ears, ear popping/clicking, resonance, distortion, and/or vibration in the ears) when exposed to loud sounds [6]. Pain hyperacusis, also called noxacusis, is characterised by the experience of aural pain (e.g., stabbing, throbbing, pinching, dull ache, sharp pain, burning pain) when exposed to certain day-to-day sounds [6,7,8]. Some individuals with pain hyperacusis also report perceiving pain in their face, sides of head, behind the ears, or elsewhere in their body [8]. Pain hyperacusis is different from the “auditory nociception” which is a term used for a pain-like sensation in the ear resulting from a damage to the hair cells or other tissues in the cochlea following exposure to unsafe levels of noise [9,10]. According to the most recent studies on the effect of noise on the human body, safe noise exposure to prevent tissue damage within the cochlea and to prevent noise-induced hearing loss is about 55–60 dB (A) for a day [11,12]. Individuals with pain hyperacusis report experiencing pain even from short-lasting sounds with intensities between 30 and 60 dB (HL) [13,14] which are well below the level that can cause any damage to the cochlea in a short span of time. Fear hyperacusis, also known as phonophobia, is defined as being afraid of exposure to certain sounds as an anticipatory fear response to either the loudness of the sounds or their impact on the individual, which can exist with or without loudness and pain hyperacusis [3,5]. In fear hyperacusis, it is the fear that a sound may occur and will either hurt their ears, make their hearing or tinnitus worse, make them more sensitive to sounds, or impact on their mental or physical health, that is the dominant symptom [5,15]. Misophonia is defined as the experience of extreme annoyance, disgust, anger, and anxiety when hearing one or more specific sound(s) or stimuli associated with such sounds [5,16,17]. The sounds that typically impact individuals with misophonia include but are not limited to sounds associated with oral functions (e.g., chewing, swallowing, lip smacking, slurping, certain people’s voice, whistles, and certain letters such as “S”), nasal sounds (e.g., breathing, snoring), non-oral/nasal sounds produced by people (e.g., tapping, clicking, crepitus), and sounds produced by objects (e.g., clock ticking) or sounds generated by animals (e.g., clucking) [16,18]. Another form of sound sensitivity that can co-occur with hyperacusis and misophonia is called “noise sensitivity” [15]. This is a personality trait characteristic involving underlying attitudes towards noise in general [19]. A person with high noise sensitivity may perceive noise caused by neighbours, nearby factories, workshops, farms, radiators, air conditioning, and background music as disruptive and distressing, regardless of the loudness of such sounds [19,20]. Noise sensitivity can sometimes be high enough to cause significant distress in a person’s life [21,22].

The Sound Sensitivity Symptoms Questionnaire version 2 (SSSQ2) is a brief self-report measure developed at Hashir International Institute (HII), United Kingdom, as a clinical tool for audiologists and other relevant healthcare professionals. The first version of this questionnaire, the SSSQ, had five items assessing loudness hyperacusis, pain hyperacusis, misophonia, and fear hyperacusis [2]. The second version, the SSSQ2, has six items. One item has been added to assess noise sensitivity (item 6). We previously reported psychometric properties for the first version of SSSQ, which was found to be a one-factor questionnaire with good internal consistency, with a Cronbach’s α of 0.87 [2,23]. However, it is necessary to test the second version in a new sample in order to establish its factor structure, validity, and reliability [24]. The SSSQ2 is intended to be used (1) as a psychometric measure for severity of sound sensitivity symptoms in general (based on its total score) and (2) as a checklist to screen different forms of sound sensitivity (i.e., loudness hyperacusis, pain hyperacusis, fear hyperacusis, misophonia, and noise sensitivity). Therefore, in addition to assessing the psychometric properties of the SSSQ2 as a six-item measure, it is important to validate the use of its individual items or combinations of the items to screen for various forms of sound sensitivity.

The aims of this study are to assess (1) the factor structure of the SSSQ2, and (2) the validity, reliability, and minimum detectable change for the SSSQ2 and its items/combinations of items.

## 2. Materials and Methods

### 2.1. Ethical Approval

The research obtained ethical approval from the University of Surrey Research Integrity and Governance Office (Project ID: FHMS 21-22 083).

### 2.2. Sound Sensitivity Symptoms Questionnaire Version 2 (SSSQ2)

As shown in Table 1, SSSQ2 is a six-item questionnaire that assesses the severity of symptoms for several types of sound sensitivity by asking the patient to indicate the frequency of occurrence of each symptom over a two weeks period. For each item, a score of 0, 1, 2, or 3 is assigned to the response categories of “0 to 1 days”, “2 to 6 days”, “7 to 10 days”, and “11 to 14 days”, respectively. The total SSSQ2 score is calculated from the sum of the scores for the six items and it ranges between 0 and 18. The SSSQ2 can be used for two purposes, namely (1) to measure the severity of sound sensitivity symptoms in general based on its total score and (2) to act as a checklist to screen different forms of sound sensitivity. The sum of items 1, 2, 3 and 5 assesses hyperacusis in general (a combination of loudness, pain and fear hyperacusis). The sum of items 1 and 3 assesses loudness hyperacusis. Item 2 asks about pain hyperacusis. Item 4 asks about misophonia. Item 5 asks about fear hyperacusis and item 6 asks about noise sensitivity.

### 2.3. Study Design and Participants

This was a psychometric study with cross-sectional survey design. An online survey was developed using the Qualtrics platform (Qualtrics, Provo, UT, USA, https://www.qualtrics.com). In addition to the SSSQ2, several validated self-report questionnaires were included in the online survey, as summarized in Table 2, comprising: the Hyperacusis Impact Questionnaire (HIQ) [2], MisoQuest [25], the Amsterdam Misophonia Scale Revised (AMISO-R) [26], the Tinnitus Impact Questionnaire (TIQ) [27,28], Screening for Anxiety and Depression in Tinnitus (SAD-T) [2], and the Hearing Handicap Inventory (HHI) [29]. The survey included two screening questions asking if the participant had tinnitus and hearing difficulties. The tinnitus question was: “Do you currently experience tinnitus that lasts more than five minutes? (Tinnitus is hearing a sound in your ears or head with no external source (e.g., buzzing, high-pitch whistle, hissing…)”. The question for hearing difficulties was: “Do you have hearing loss? (Hearing loss refers to partial or complete inability to hear sounds in one or both ears.)”. The participants who answered “yes” to the screening questions about tinnitus and hearing difficulties were directed to complete the TIQ and HHI, respectively. If they answered “no”, then they were not asked to complete the TIQ or HHI.

The survey was emailed to students and staff members at the University of Surrey and was shared with various patient support groups relevant to tinnitus and sound intolerance via email and social media platforms. Study participants were invited to complete the survey twice with a two-week interval so the data could be used for both factor analysis and assessment of test–retest reliability of the SSSQ2.

A total of 451 people completed the online survey, of which 154 individuals completed the survey twice.

### 2.4. Statistical Analysis

The data were anonymized prior to conducting statistical analyses. The structural validity of the six-item SSSQ2 was tested by confirmatory factor analysis (CFA) for which the values required for good model fit comprise: Standardized Root Mean Square Residual (SRMR) ≤ 0.08 [30], Root Mean Square Error of Approximation (RMSEA) ≤ 0.08 [30], Tucker Lewis Index (TLI) ≥ 0.90 [31], Comparative Fit Index (CFI) ≥ 0.90 [31], Goodness of Fit Indexes (GFI) ≥ 0.90 [32], and CMIN (Chi-square/df) = 3–5 [33].

The endorsement rate, which is the percentage of respondents who gave the same response for each of the SSSQ2 items, was reported. This is used to compare the frequency of experiencing various forms of sound sensitivity symptoms. Internal consistency was measured with Cronbach’s *α* and McDonald’s *ω* [34] for which values greater than 0.7 are considered acceptable [35]. Test–retest reliability over a two-week interval was determined by computing the intra-class correlation coefficient (ICC). The ICC is a value between 0 and 1, where values below 0.5 indicate poor reliability, between 0.5 and 0.75 moderate reliability, between 0.75 and 0.90 good reliability, and values above 0.90 indicate excellent reliability [36]. T-tests were used for comparing means between test and retest scores. The ICC was calculated for the total SSSQ2 score and for its items and combination of items. The minimum detectable change (MDC) was calculated using the standard error of measurement (SEM) based on the standard deviation (SD) of the difference between the test and retest scores. The MDC indicates the minimum change in the questionnaire score (i.e., total SSSQ2 score, its items and combinations of the items) that is required to be 95% confident that the difference in scores between multiple tests reflects a true change and not the measurement error [37]. The SEM and MDC were calculated with the following formulae: SEM = SD/√2 and MDC (with 95% confidence interval) = SEM × √2 × 1.96. 

The convergent and discriminant validity of the SSSQ2 were examined by calculating the Pearson correlation coefficients between SSSQ2 variables (i.e., total score, the score of the SSSQ2 items and combinations of items) and (1) the measures related to sound sensitivity construct (i.e., HIQ, MisoQuest, and AMISOS-R) for convergent validity and (2) the measures assessing constructs other than sound sensitivity, namely hearing loss, tinnitus impact, and anxiety/depression (i.e., HHI, TIQ, and SAD-T), for discriminant validity. The correlation coefficients of ≥0.5, 0.30–0.49, and 0.10–0.29 indicate strong, moderate, and weak relationships, respectively [38]. The data collected from the 451 participants who completed the first survey were used for the CFA, Cronbach’s *α* and McDonald’s *ω*, and the Pearson correlations for convergent and discriminant validity. The data for the 154 participants who completed the first and second surveys with a two-week interval were used to calculate ICC for test–retest reliability and MDC. Statistical analyses were conducted using IBM SPSS software version 28.0, and Amos version 28.0. Some of the participants did not complete all of the questionnaires included in the survey. The number of participants (N) who were included to each analysis is reported.

## 3. Results

### 3.1. Characteristics of the Study Population

The average age of the participants was 36.5 years (standard deviation, SD = 12.8 years, range 18 to 86 years) and 60.3% were female. The mean scores and SD for the SSSQ2, MisoQuest, HIQ, AMISOS-R, SAD-T, TIQ, and HHI were 6.6 (SD = 4.3, N = 451), 49 (SD = 12, N = 451), 9.6 (SD = 6.1, N = 451), 18.8 (SD = 9.2, N = 451), 2.4 (SD = 1.9, N = 448), 7.7 (SD = 6.0, N = 173), 21.2 (SD = 9.4, N = 87), respectively. Only participants who reported having hearing difficulties (18.4% of the sample, N = 87) completed the HHI. Based on the scores for the HHI, among patients with self-reported hearing difficulties, 11.5% (N = 10) had no hearing handicap, 51.7% (N = 45) had a mild-moderate handicap, and 36.8% (N = 32) had a severe hearing handicap. Based on scores for the HIQ, hyperacusis had a significant impact among 35.5% of patients (N = 160). Based on scores for MisoQuest, 21.7% of patients (N = 98) had significant misophonia. Based on scores for the AMISOS-A, 20.2% of patients (N = 91) had no misophonia (i.e., had sub-clinical symptoms), 34.1% (N = 154) had a mild misophonia, 35.5% (N = 160) had a moderate misophonia, and 10.2% (N = 46) had a severe to extreme misophonia. Based on scores for the SAD-T, 27% of patients (N = 121) had symptoms of anxiety and/or depression. Thirty-nine percent of patients (N = 176) reported experiencing tinnitus, of which 173 completed the TIQ. Based on scores for the TIQ, 36.4% of patients (N = 63) had no tinnitus handicap, 9.3% (N = 16) had a mild tinnitus handicap, 12.1% (N = 21) had a moderate tinnitus handicap, and 42.2% (N = 73) had a severe tinnitus handicap.

### 3.2. Confirmatory Factor Analysis

**Figure 1:**
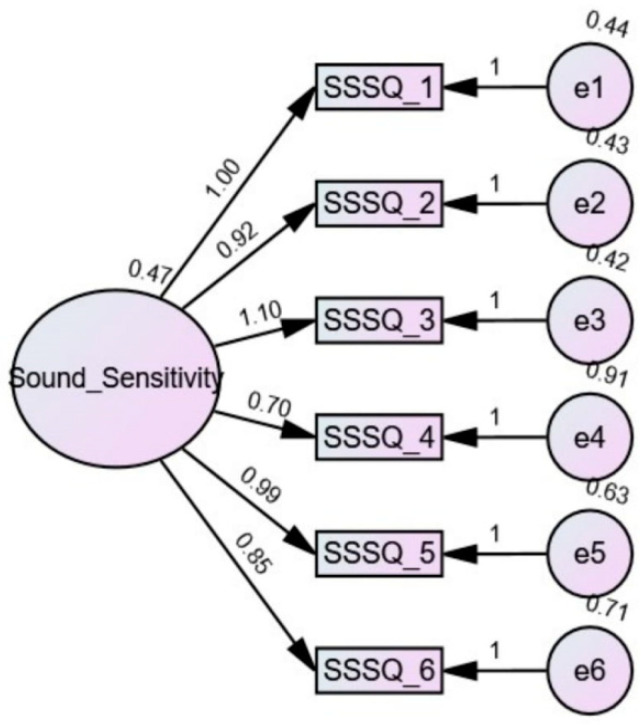
The factor structure of the SSSQ2.

The factor structure of the SSSQ2, is shown in Figure 1. One factor model for the SSSQ2 with six items gave a good fit for all measures of goodness of fit. Table 3 demonstrates that RMSEA and SRMR values met the criteria of ≤0.08, and CFI, TLI and GFI of ≥0.90. The CMIN was very close to 3. These findings support a good fit for the one factor model for the SSSQ2. Table 4 shows that item 6 (noise sensitivity) and item 4 (misophonia) were endorsed more than other items of the SSSQ2. With regard to item endorsement, items 6 and 4 were followed by items 1, 5, 3, and 2. Item 2 (pain hyperacusis) was the least endorsed item. The higher percentage of people selected the response choice of “2–6 days” for items 1, 4, and 6. However for items 2, 3, and, 5, the most frequently selected response choice was “0–1 days”.

### 3.3. Reliability

Both Cronbach’s α and McDonald’s *ω* estimate for the SSSQ2 were 0.80, which indicate acceptable internal consistency. All of the items were strongly correlated with the total SSSQ2 score (Table 5). As shown in Table 6, there were no statistically significant differences in the mean scores for SSSQ2 variables between the first and the second surveys, which were taken with a two-week interval (except from the score for item 5, fear hyperacusis). The score for item 5 was slightly but significantly lower in the second test (retest) compared with the first test by an average of 0.29 points. However, the mean change for other SSSQ2 variables between the first and second tests was less than 0.28 points and not statistically significant. The test–retest reliability was rated as good for the total SSSQ2 score (indicating the severity of sound sensitivity symptoms) and the sum of items 1, 2, 3, and 5 (indicating general hyperacusis) based on their ICC values, which were above 0.75. The test–retest reliability was rated as moderate for the sum of items 1 and 3 (indicating loudness hyperacusis) based on the ICC value of between 0.5 and 0.75. Single items comprising: item 2 (for pain hyperacusis), item 4 (for misophonia), item 5 (for fear hyperacusis), and item 6 (for noise sensitivity) also shown to have moderate test–retest reliability.

Based on the MDC values, when this questionnaire is used for repeated measurements, the minimum amount of change that constitutes a true change in sound sensitivity is ≥5 for the total SSSQ2 score, ≥4 for general hyperacusis (the sum of items 1, 2, 3, and 5), ≥3 for loudness hyperacusis (sum of items 1 and 3), and ≥2 for pain hyperacusis (item 2), misophonia (item 4), fear hyperacusis (item 5), and noise sensitivity (item 6) (Table 6).

### 3.4. Discriminant and Convergent Validity

Discriminant validity of the total SSSQ2 score was demonstrated based on its weak relationship with the HHI score. The scores for general hyperacusis (the sum of items 1, 2, 3 and 5), loudness hyperacusis (sum of items 1 and 3), pain hyperacusis (item 2), misophonia (item 4), fear hyperacusis (item 5), and noise sensitivity (item 6) were also weakly correlated with the score on HHI demonstrating that they too differentiate between handicap caused by hearing loss and various forms of sound sensitivity. The weakest relationship was observed between misophonia (item 4) and HHI (Table 7).

The total score for the SSSQ2 (sum of all six items), and the scores for general hyperacusis (sum of items 1, 2, 3, and 5), and loudness hyperacusis (sum of items 1 and 3) were strongly correlated with the total HIQ score. The scores for pain hyperacusis (item 2), misophonia (item 4), fear hyperacusis (item 5), and noise sensitivity (item 6) were moderately with total HIQ score. Overall, this provides evidence for convergent validity (Table 7). The misophonia score (item 4) was moderately correlated with the MisoQuest and AMISOS-R scores, which are self-report questionnaires also aiming to measure the construct of misophonia. The total SSSQ2 score was moderately and strongly correlated with MisoQuest and AMISOS-R, respectively. This shows that SSSQ2 encompasses the construct of misophonia in addition to hyperacusis. It is worth noting that the scores for general hyperacusis (sum of items 1, 2, 3, and 5), loudness hyperacusis (sum of items 1 and 3), pain hyperacusis (item 2), fear hyperacusis (item 5), and noise sensitivity (item 6) were weakly correlated with the total MisoQuest score, providing evidence for their discriminant validity as they aim to assess the constructs that are different from misophonia (i.e., hyperacusis and noise sensitivity). As shown in Table 7, correlations with another misophonia questionnaire (AMISOS-R) showed that except from the score of pain hyperacusis (item 2), which was weakly correlated with the AMISOS-R, the scores of general hyperacusis (sum of items 1, 2, 3, and 5), loudness hyperacusis (sum of items 1 and 3), fear hyperacusis (item 5), and noise sensitivity (item 6) were moderately correlated with the AMISOS-R total scores. There were moderate to strong correlations between SSSQ2 and the scores of SAD-T and TIQ indicating that severity of sound sensitivity in general and various forms of sound sensitivity are related to mental health and tinnitus distress (among patients who also had tinnitus). There was a weak but not statistically significant correlation between SSSQ2 and age. The total SSSQ2 score was not significantly correlated with gender (*r* = 0.004, *p* = 0.93).

## 4. Discussion

Our results showed that the SSSQ2 can be used as a one-factor questionnaire to assess the construct of sound sensitivity symptoms encompassing various forms of hyperacusis (i.e., loudness, pain, and fear hyperacusis), misophonia, and noise sensitivity. Therefore, the total SSSQ2 score can be used in day-to-day clinical practice or research settings to measure the severity of sound sensitivity as a whole. This is consistent with previous studies reporting a significant overlap between symptoms of various forms of hyperacusis, misophonia, and noise sensitivity [6,39,40,41]. The total SSSQ2 score is strongly correlated with the HIQ and AMISOS-R scores and moderately correlated with the total MisoQuest score. These questionnaires aim to assess constructs related to sound sensitivity (i.e., the HIQ assesses the impact of hyperacusis on the patient’s life, while AMISOS-R and MisoQuest assess the severity of misophonia symptoms) so their relationships with SSSQ2 indicate the convergent validity of the SSSQ2. The total SSSQ2 score was only weakly related to the HHI scores. This indicates the discriminant validity of the SSSQ2 because the HHI assesses the impact of hearing impairment which is a construct different from that of sound sensitivity. Past research suggests that hyperacusis and misophonia are not related to hearing impartment [42,43]. The total SSSQ2 score was strongly correlated with anxiety and depression as measured via SAD-T and tinnitus distress as measured via TIQ. This is consistent with previous research studies suggesting that hyperacusis, misophonia, and noise sensitivity are closely linked with anxiety and depression as well as tinnitus distress (among patients with tinnitus) [44,45,46]. The total SSSQ2 score was weakly but not significantly correlated with age. This is different from a small but statistically significant negative correlation reported between the score of the first version of the SSSQ and age [2]. The discrepancy with regard to the relationship with age may be due to the characteristics of the study populations. The study population in the present study was younger with the mean age of was 36.5 years (SD = 12.8 years) compared to that of the study on the first version of the SSSQ with the mean age of 54 years (SD = 16 years).

It is important to note that although all six items of the SSSQ2 are strongly correlated with its total score (as shown in Table 5), it is possible that a patient reports very frequent experience of a particular form of sound sensitivity (i.e., by selecting the response choice of “11–14 days” for a single item) without exhibiting high scores on other items. This can lead to a relatively low total SSSQ2 score, despite reporting high severity for one form of sound sensitivity (e.g., only reporting misophonia without any hyperacusis or noise sensitivity components). Therefore, the clinicians and researchers should pay attention to the scores of individual items too (e.g., item 2 for pain hyperacusis, item 4 for misophonia, item 5 for fear hyperacusis and item 6 for noise sensitivity). Our results showed that the total SSSQ2 score (sum of the six items) and the score for general hyperacusis (sum of items 1, 2, 3, 5) have good test–retest reliability with ICC values of above 0.75. However, when using the score for loudness hyperacusis (sum of items 1 and 2), or single items for pain hyperacusis (item 2), misophonia (item 4), fear hyperacusis (item 5) and noise sensitivity (item 6), the test–retest reliability is at moderate level with ICC values between 0.50 and 0.75. As the test–retest reliability of the single items is less than that of the total SSSQ2 score, single items should be used with caution, taking this limitation into account.

The SSSQ2 can be used in clinical practice or research to assess the change in self-report severity of sound sensitivity symptoms before and after a treatment. In the present study, we reported the values of MDC, which demonstrate the minimum change in the questionnaire score that is required to reflect a true change beyond the measurement error of the questionnaire when used for repeated measurements [37]. However, the MDC should be differentiated from minimally important change (MIC), which represents changes in questionnaire scores which are considered to be minimally important by healthcare professionals and patients [47]. In other words, the changes in questionnaire scores exceeding the MDC reported in this study indicate true changes in the scores but they do not necessarily represent a clinically relevant change. Future studies should assess the MIC values for the SSSQ2, for which anchor-based methods can be used [48]. For instance, a global rating of change [49] can be used to assess patients’ and clinicians’ views with regard to whether the severity of sound sensitivity has got worse, better or stayed the same and to quantify the magnitude of that change following a treatment. The global rating of change can be used as the anchor to assess which changes on the score of the SSSQ2 corresponds with a MIC defined on the anchor.

As the data collection in this study was via an online survey, hearing thresholds and uncomfortable loudness levels (ULLs) were not measured. This is a limitation of the data, as they were solely dependent on self-report measures. Future studies should compare pure-tone hearing thresholds and ULLs among participants with different forms of sound sensitivity as measured via the SSSQ2. Another limitation is that the study population was largely non-clinical, hence the performance of the SSSQ2 when used for assessing patients seeking help for sound sensitivities require more research in a clinical population. The first version of the SSSQ has shown good internal consistency when tested in a clinical population [23], however, as the SSSQ2 has an additional item, a reassessment of its psychometric properties in a clinical population is required.

## 5. Conclusions

The total SSSQ2 score can be used in day-to-day clinical practice or research settings to measure the severity of general sound sensitivity as a one-factor questionnaire (based on the results of the CFA) with acceptable internal consistency (based on the values of Cronbach’s α and McDonald’s *ω*). The test–retest reliability of the SSSQ2 is good based on ICC analysis. The total SSSQ2 score ranges between 0 and 18. The minimum change that reflects a true change in SSSQ2 total scores is a change of five points or more based on the value of MDC. Future research should determine the amount of change in SSSQ2 scores that can constitute as a clinically important change following a treatment. The SSSQ2 can be used a checklist to screen for various forms of sound sensitivity comprising: loudness hyperacusis (sum of items 1 and 2), pain hyperacusis (item 2), misophonia (item 4), fear hyperacusis (item 5), and noise sensitivity (item 6). The test–retest reliability of the single items is moderate (less than that of the total SSSQ2 score), so their scores for screening of different forms of sound sensitivity should be interpreted with caution. The SSSQ2 and its items have shown good convergent and discriminant validity.

## Figures and Tables

**Table 1:** Items and response choices of the Sound Sensitivity Symptoms Questionnaire version 2 (SSSQ2).

Over the Last 2 Weeks, How Often Have You Been Bothered by Any of the Following Problems?				
1. Having a problem tolerating sounds because they often seem “too loud” to you?	0–1 days	2–6 days	7–10 days	11–14 days
2. Pain in your ears when hearing certain loud sounds? *Examples: loud music, sirens, motorcycles, building work, lawn mower, train stations.*	0–1 days	2–6 days	7–10 days	11–14 days
3. Discomfort (physical sensations other than ear pain) in your ears when hearing certain loud sounds?	0–1 days	2–6 days	7–10 days	11–14 days
4. Feeling angry or anxious when hearing certain sounds related to eating noises, lip smacking, sniffling, breathing, clicking sounds, tapping?	0–1 days	2–6 days	7–10 days	11–14 days
5. Fear that certain sounds may make your hearing and/or tinnitus worse?	0–1 days	2–6 days	7–10 days	11–14 days
6. Getting disturbed because of general environmental noise (e.g., noise in your neighbourhood, nearby airports and industrial facilities, distant traffic sound, noisy pipes or cracking noises in the house, air conditioning, etc.)?	0–1 days	2–6 days	7–10 days	11–14 days

**Table 2:** The number of items, scoring, and internal reliability (as measured via Cronbach’s α) of the self-report questionnaires used in the online survey in addition to the SSSQ2.

Name	Number of Items	Scoring	Cronbach’s Alpha
Hyperacusis Impact Questionnaire (HIQ)	8	Total score ranges from 0 to 24. HIQ score > 11 shows presence of clinically significant hyperacusis impact.	0.91
MisoQuest	14	The total score ranges from 0 to 70. Scores > 61 indicate presence of misophonia.	0.93
Amsterdam Misophonia Scale Revised (AMISO-R)	10	Total score ranges between 0 and 40. Scores < 10 = subclinical symptoms, between 11 and 20 = mild, between 21 and 30 = moderate and >30 equals severe to extreme misophonia symptoms.	0.92
Hearing Handicap Inventory (HHI)	10	Total score ranges from 0 to 40. Scores between <8 indicate no hearing handicap; 10–24 mild-moderate handicap; and >26 indicate severe handicap.	0.84
Tinnitus Impact Questionnaire (TIQ)	7	The overall score ranges from 0 to 21. A score < 5 indicates no impact of tinnitus, a score of 5 or 6 indicates mild impact, a score of 7 or 8 indicates moderate impact, and a score of >9 indicates a severe impact.	0.89
Screening for Anxiety and Depression in Tinnitus (SAD-T)	4	Total score ranges from 0 to 12. SAD-T score ≥ 4 shows possible anxiety and/or depression.	0.87

**Table 3:** Goodness-of-fit indices for the SSSQ2.

Fit Statistics	One Factor Model Result from CFA
CMIN (χ^2^/df)	2.970
RMSEA	0.066
CFI	0.982
TLI	0.962
GFI	0.985
SRMR	0.031

Note. SSSQ2: Sound Sensitivity Symptoms Questionnaire version 2; CMIN: Chi-square/df; RMSEA: root mean squared error of approximation; CFI: comparative fit index; TLI: Tucker–Lewis index; GFI: Goodness of fit indexes; SRMR: standardized root mean squared residual.

**Table 4:** Endorsement rates (%) for each item of the SSSQ2 (N = 451).

Item	Endorsement Rates % (N)			
	0–1 Days	2–6 Days	7–10 Days	11–14 Days
1	29.9 (135)	38.4 (173)	21.3 (96)	10.4 (47)
2	41.5 (187)	34.8 (157)	17.3 (78)	6.4 (29)
3	40.6 (183)	29.7 (134)	20.2 (91)	9.5 (43)
4	28.4 (128)	31.9 (144)	21.5 (97)	18.2 (82)
5	42.6 (192)	25.9 (117)	19.5 (88)	12.0 (54)
6	24.8 (112)	36.8 (166)	21.5 (97)	16.9 (76)

Note. SSSQ2: Sound Sensitivity Symptoms Questionnaire version 2, N: number of participants.

**Table 5:** Correlations between the total SSSQ2 score, its individual items and the combinations of the items (N = 451). Numbers in the table refer to the correlation coefficients (*r*). All of the correlations were statistically significant (*p* < 0.01). The total SSSQ2 score represents the severity of sound sensitivity symptoms. The sum of the items 1, 2, 3 and 5 (Items 1235) represents hyperacusis in general. The sum of items 1 and 3 (items 13) represents loudness hyperacusis. Item 2 represents pain hyperacusis. Items 4, 5, and 6 represent misophonia, fear hyperacusis, and noise sensitivity, respectively.

	Item 1	Item 2	Item 3	Item 4	Item 5	Item 6	Items 1235	Items 13	Total SSSQ2
Item 1	1	0.46	0.55	0.37	0.42	0.49	0.77	0.88	0.77
Item 2	0.46	1	0.53	0.24	0.53	0.39	0.79	0.57	0.72
Item 3	0.55	0.53	1	0.34	0.52	0.39	0.82	0.89	0.78
Item 4	0.37	0.24	0.34	1	0.27	0.34	0.38	0.40	0.61
Item 5	0.42	0.53	0.52	0.27	1	0.31	0.79	0.53	0.72
Item 6	0.49	0.39	0.39	0.34	0.31	1	0.50	0.50	0.68
Items 1235	0.77	0.79	0.82	0.38	0.80	0.49	1	0.90	0.94
Items 13	0.88	0.57	0.89	0.40	0.53	0.50	0.90	1	0.87
Total SSSQ2	0.77	0.72	0.78	0.61	0.72	0.68	0.94	0.87	1

Note: SSSQ2: Sound Sensitivity Symptoms Questionnaire version 2; N: number of participants included to the analysis; *p*: *p*-value.

**Table 6:** The table shows the means (M) and standard deviations (SD) for the SSSQ2 variables calculated for the first and the second times that participants completed the survey for assessment of test–retest reliability (N = 154). In addition, the table shows the Interclass Correlation Coefficient (ICC) values and their 95% Confidence Intervals (CI), the Standard Error of Measurement (SEM) and the Minimum Detectable Change (MDC) separately calculated for (a) severity of sound sensitivity symptoms (via the total SSSQ2 score, i.e., the sum of the six items), (b) General Hyperacusis (the sum of items 1, 2, 3 and 5), (c) Loudness Hyperacusis (sum of items 1 and 3), (d) Pain Hyperacusis (item 2), (e) Misophonia (item 4), (f) Fear Hyperacusis (item 5), and (g) Noise Sensitivity (item 6).

	First Test M (SD)	Retest M (SD)	t p 95% CI	ICC [95% CI]	SEM	MDC
SSSQ2 Total	6.75 (4.0)	6.48 (4.35)	0.92 0.36 [−0.31,0.85]	0.81 [0.73, 0.86]	1.74	4.82
General Hyperacusis	3.94 (2.98)	3.76 (3.11)	0.75 0.45 [−0.29, 0.64]	0.76 [0.66, 0.83]	1.46	4.04
Loudness Hyperacusis	2.08 (1.80)	2.19 (1.81)	−0.67 0.50 [−0.42, 0.20]	0.66 [0.53, 0.76]	1.05	2.91
Pain Hyperacusis	0.85 (0.94)	0.84 (1.02)	0.08 0.93 [−0.18, 0.20]	0.53 [0.34, 0.67]	0.64	1.77
Misophonia	1.41 (1.09)	1.33 (1.052)	0.81 0.42 [−0.11, 0.26]	0.66 [0.52, 0.76]	0.64	1.77
Fear Hyperacusis	1.02 (1.08)	0.73 (0.99)	3.16 0.02 [0.11, 0.47]	0.64 [0.49, 0.74]	0.65	1.80
Noise Sensitivity	1.48 (1.09)	1.39 (1.07)	0.87 0.38 [−0.11, 0.27]	0.65 [0.50, 0.75]	0.64	1.78

Note. SSSQ2: Sound Sensitivity Symptoms Questionnaire version 2; N: number of participants; *p*: *p*-value; t: t statistics with degree of freedom equal to 129.

**Table 7:** Correlation coefficients (*r*) calculated separately between the scores for various SSSQ2 variables (i.e., Sound Sensitivity Symptoms, General Hyperacusis, Loudness Hyperacusis, Pain Hyperacusis, Misophonia, Fear Hyperacusis, and Noise Sensitivity as explained in Table 6) and HIQ, SAD-T, HHI, MisoQuest, AMISOS-R, TIQ, and age.

	HIQ N = 451	SAD-T N = 447	HHI N = 87	MisoQuest N = 451	AMISOS-R N = 451	TIQ N = 173	Age N = 451
SSSQ2 Total	0.73 *p* < 0.01	0.56 *p* < 0.01	0.21 *p* = 0.11	0.31 *p* < 0.01	0.50 *p* < 0.01	0.59 *p* < 0.01	0.04 *p* = 0.78
General Hyperacusis	0.68 *p* < 0.01	0.51 *p* < 0.01	0.22 *p* = 0.05	0.20 *p* < 0.01	0.41 *p* < 0.01	0.61 *p* < 0.01	0.06 *p* = 0.72
Loudness Hyperacusis	0.68 *p* < 0.01	0.46 *p* < 0.01	0.18 *p* = 0.14	0.26 *p* < 0.01	0.40 *p* < 0.01	0.51 *p* < 0.01	0.07 *p* = 0.33
Pain Hyperacusis	0.44 *p* < 0.01	0.34 *p* < 0.01	0.13 *p* = 0.35	0.058 *p* = 0.05	0.24 *p* < 0.01	0.44 *p* < 0.01	0.05 *p* = 0.85
Misophonia	0.48 *p* < 0.01	0.42 *p* < 0.01	0.01 *p* = 0.85	0.46 *p* < 0.01	0.47 *p* < 0.01	0.32 *p* < 0.01	−0.07 *p* = 0.06
Fear Hyperacusis	0.48 *p* < 0.01	0.41 *p* < 0.01	0.27 *p* = 0.02	0.08 *p* = 0.02	0.30 *p* < 0.01	0.59 *p* < 0.01	−0.01 *p* = 0.48
Noise Sensitivity	0.50 *p* < 0.01	0.40 *p* < 0.01	0.13 *p* = 0.29	0.22 *p* < 0.01	0.34 *p* < 0.01	0.36 *p* < 0.01	−0.03 *p* = 0.76

Note. SSSQ2: Sound Sensitivity Symptoms Questionnaire version 2; HIQ: Hyperacusis Impact Questionnaire; SAD-T: Screening for Anxiety and Depression in Tinnitus; HHI: Hearing Handicap Inventory; AMISOS-R: Amsterdam Misophonia Scale Revised; TIQ: Tinnitus Impact Questionnaire; N: number of participants; *p*: *p*-value.

## Data Availability

The raw data supporting the conclusions of this article will be made available by the authors upon request.
